# Current and future prospects for nanotechnology in animal production

**DOI:** 10.1186/s40104-017-0157-5

**Published:** 2017-03-14

**Authors:** Emily K. Hill, Julang Li

**Affiliations:** 1 School of Life Science and Engineering, Foshan University, Foshan, Guangdong China; 2 Department of Animal Biosciences, University of Guelph, 50 Stone Road East, Building #70, Guelph, ON N1G 2 W1 Canada

**Keywords:** Animal production, Antibiotic replacements, Artificial insemination, Biocides, Feed, Nanoparticles, Veterinary medicine

## Abstract

Nanoparticles have been used as diagnostic and therapeutic agents in the human medical field for quite some time, though their application in veterinary medicine and animal production is still relatively new. Recently, production demands on the livestock industry have been centered around the use of antibiotics as growth promoters due to growing concern over microbial antibiotic resistance. With many countries reporting increased incidences of antibiotic-resistant bacteria, laws and regulations are being updated to end in-feed antibiotic use in the animal production industry. This sets the need for suitable alternatives to be established for inclusion in feed. Many reports have shown evidence that nanoparticles may be good candidates for animal growth promotion and antimicrobials. The current status and advancements of nanotechnological applications in animal production will be the focus of this review and the emerging roles of nanoparticles for nutrient delivery, biocidal agents, and tools in veterinary medicine and reproduction will be discussed. Additionally, influences on meat, egg, and milk quality will be reviewed.

## Background

**Fig. 1:**
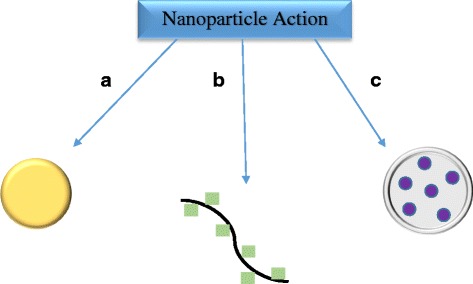
Three basic systems of nanoparticles in their applications. Nanoparticles can serve as the functional unit (a) but can also act as a delivery vehicle for materials conjugated to their surface (b) or encapsulated within (c)

Nanotechnology is the study of materials at the nanoscale. With at least one dimension generally ranging between 1 and 100 nm (10^−9^ – 10^−7^m), nanomaterials are best referred to as particles [1, 2]. These nanoparticles are particularly appealing as they take up very little space yet have relatively large surface areas, and therefore an increased ratio between surface atoms and interior atoms. As a result, when bulky materials are scaled down to nanosizes, their surface chemistries become more influential and alter the physical properties of the material [2]. For example, copper is known for its malleability, a useful feature for wiring and piping. However, when copper is scaled down into a nanoform, it loses its malleability as its surface atoms resist bending [3]. The interior copper atoms in a bulkier form facilitate bending but are out-numbered by surface atoms in the nanoform. Enlarging the ratio of surface area to volume allows for nanoparticles to be more versatile, whether as a single functional unit, or as a carrier for functional units which can be adhered to their surfaces or encapsulated within (Fig. 1). Nanoparticles are becoming more attractive as novel uses, from medical diagnostics to gene therapy vehicles, are discovered.

## Types of nanoparticles

Nanoparticles, currently available or under development, can be categorized into four groups: metals, polymers, natural compounds, and nanostructured materials. Although different engineering techniques are required depending on the group, nanoparticles can facilitate an array of biotechnical functions through different mechanisms of action (Table 1). Metal nanoparticles are the powdery version of solid metal, after large pieces have been ground down to nanosizes, effectively changing its associated physical properties [4]. These particles have drawn the attention of the medical field for their use in imaging and as antimicrobial therapies that lyse Gram positive and Gram negative bacterial cell walls [5]. External or topical applications may be more suitable for some metal nanoparticles to avoid accumulation in the body, as certain species can elicit harmful dosage-toxicity responses, although this is not always the case [6–8]. The non-biodegradable nature of metal is a major drawback for these particles. Polymeric nanoparticles, or nano-polymers, are polymers that have been synthesized or fragmented into pieces that are nanometers long. Nano-polymers have the ability to be grafted onto other materials, potentially improving their biocompatibility and degradation while expanding their utility [8]. Biocompatibility is highly advantageous for the medical and food industries as working concentrations of biocompatible nanoparticles will have few to no negative side effects on patients or consumers [9–12]. Similar to the metal varieties, polymeric nanoparticles with a fluorescent or radiolabeled component may be used for medical imaging, although dosage toxicity would still have to be considered [13]. Nanoparticles made of natural compounds are materials that come from nature with limited manipulation, such as natural polymers or proteins. With few alterations, natural compounds are more likely to be biocompatible, distributable in the body, and biodegradable. Nanostructured materials are synthesized nanoparticles that originate from many sources, including natural compounds such as lipid- and protein-based nanoparticles. Natural and nanostructured nanoparticles share many advantages and can serve as the sole functioning unit or carriers for functional groups, such as drugs and nutrients, via encapsulation or superficial adhesion. While nature-derived nanomaterials may seem a safer choice, these particles could elicit toxic or immunogenic responses if not carefully engineered or appropriately distributed in a biological system. Despite these potential limitations, the advantages of employing nanotechnology are far greater.

**Table 1:** Mechanisms of action for nanoparticles performing a function useful to animal production

Function	Type of Nanoparticle	Mechanism of Action	References
Nutriceuticals	Metal	- feed supplements at the nanoscale are more bioavailable to animals than at a microscale, allowing more interactions to occur in the gut and better absorbance	[27, 38, 47, 51, 52]
	Natural	- nanoparticle additives to food products for human consumption can increase bioavailability	[55]
Drug and Nutrient Delivery	Polymer	- can be loaded with traditional antibiotics and may act as a shuttle to release them when in close proximity to a pathogen	[41–43]
		- metal nanoparticles may be conjugated to polymers for a combined nutrient/biocide delivery approach	[48, 49]
	Natural	- enclose around nutrients to protect against their degradation in the stomach for maximum intestinal absorption	[21]
	Nanostructured	-designed to carry nutrients or pharmaceuticals through the gastrointestinal tract for targeted release	[26, 66, 67]
Biocides	Metal	- lyse negatively charged Gram + and Gram – bacterial cell walls	[5, 31, 32, 53]
	Polymer	- destabilize bacterial cell walls such that homeostasis is disrupted to a lethal extent	[33, 44, 45]
Diagnostic Tools	Metal	- magnetic metal nanoparticles can disperse throughout the body and be imaged via MRI	[60]
	Nanostructured	- fluorescence can be initiated via light activation or two-photon excitation	[61, 62, 65]
Reproductive Aids	Nanostructured	- purification of sperm through the removal of damaged spermatozoa via surface markers recognized by nanoparticle-bound antibodies or lectins	[75, 76]
Molecular Biology Agents	Nanostructured	- gene transfer mediation through interactions between nucleic acids, nanoparticles, and sperm	[81]
	Polymer	- DNA transfection vehicles (as above)	[82]

## Applications of nanotechnology

Current applications of nanotechnology in the medical and food industries are relatively analogous. Medical diagnostics and food safety testing are being improved through lab-on-a-chip technologies, capitalizing on the large surface area and small volume of nanoparticles. These molecular technologies require less samples, shorter run times, and provide a simpler user experience without the need for large bench top machines [14, 15]. A major advantage of lab-on-a-chip technologies is the ability to generate real-time data in the field. Therapeutic nanoparticles and nanoparticles as therapy delivery systems are both growing interests in the medical field and parallel the food industry where nanoparticles are being investigated as nutraceutical delivery systems, and biocides to better preserve food stuffs. Germicidal metal and polymeric nanoparticles attack the integrity of microbial cell walls, while natural and nanostructured materials can entrap and protect nutrients for delivery. The complementary nature of the medical and food industries is best exemplified in animal production where livestock health is directly linked to food safety. Livestock producers require their herds and flocks to reach ideal slaughter weights rapidly to maximize profitability. To achieve this, antibiotics are currently used prophylactically as feed additives to prevent illness and accelerate growth, thus shortening animal production cycles [16]. While helpful from the production standpoint, this global practice has led to the rise of drug-resistant bacteria that can cause illness in livestock and contaminate meat [17]. Trends in antibiotic use from various countries show that the majority is used in agriculture and less in humans [18]. This has prompted several countries to pass legislation restricting the use of antibiotics in animal production [19]. Nanoparticles not only have the potential to fill the gap created by these restrictions, but can also do so without driving antibiotic-resistance in microbes. Further to this, nutrient delivery, biocides, veterinary medicine, and reproduction are areas of the animal production industry that could benefit from nanotechnology, and the advantages and limitations of their inclusion will be discussed in this review (Table 2).

**Table 2:** Current and future applications of nanotechnology in animal production with their advantages and limitations

	Advantages	Limitations	References
Current applications			
Medical diagnostics	- disease diagnosis and prognosis - small sample volume requirements - faster sample analyses; faster action time - suitable for field use	- greater sample preparation measures - sensitivity concerns from small sample sizes - device pricing	[14, 15, 60–64]
Medical supplies	- antibacterial wound dressings - prevention of catheter-caused infections - inhibits biofilm formation	- patient sensitivities and allergies - potential free radical formation	[85, 86]
Food safety	- contaminant and pathogen identification and indication - contamination prevention	- food and food packaging safety concerns	[53, 55]
Biocides	- alternatives to antibiotics and conventional cleaners - internal and external uses - antimicrobial coatings - multi-purpose (i.e. bactericidal and growth promoting)	- little in vivo evidence to support many internal applications demonstrated in vitro - cytotoxicity	[31–34, 44, 45, 85, 86]
Future applications			
Nutraceuticals	- increased nutrient bioavailability - extra support for weanlings - growth and performance enhancement - solution for nutrient deficiencies	- nanoparticles must be not be degraded in the GI tract before absorption in the intestines	[27, 38, 47–52, 59]
Nutrient delivery systems	- increased nutrient bioavailability - extra support for weanlings - growth and performance enhancement	- carrier system must be designed to withstand GI tract challenges - bioaccumulation - biocompatibility concerns	[21, 26]
Therapeutics	- alternatives to antibiotics - enhanced pathogen/organ targeting	- biocompatibility considerations - efficacy against different pathogens - relative MIC	[41–43, 53]
Drug delivery systems	- platforms to enhance drug specificity and delivery - reduce MIC and kill antibiotic-resistant strains of pathogens	- biocompatibility considerations - potential for bodily accumulation - confidence in specificity	[61, 62, 65–67]
Medical Imaging	- additional modes of tracing organ systems and tracking drug therapies in the body	- depth of tissue - biocompatibility considerations	[61, 62, 65]
Nanopurification of sperm	- isolate desired sperm based on biomarkers (i.e. healthy from unhealthy) - increase fertilization efficiency, more females fertilized from a single collection	- biomarker library to be developed - purebred restrictions on artificial insemination	[75, 76, 83, 84]
Cryopreservation of sperm	- sperm preserved for longer - protecting against freeze-thaw cycles - to replace antibiotics from extenders; lower risk of reduced sperm motility	- spermatotoxicity - oocyte toxicity	[76–80, 83, 84]
Genetic manipulation	- can carry DNA of interest from cytoplasm into cell nucleus - benefits to sperm-mediated gene transfer approach - transduction replacement strategy (no viruses involved in DNA administration)	- cytotoxicity - must not disturb cellular functioning	[81–84]

## Nutrient delivery

Casein micelles are naturally occurring nanoparticles in milk, where casein phosphoproteins make up approximately 80% of the protein profile in cow’s milk [20, 21]. Some casein isoforms assemble themselves around calcium, proteins, and other nutrients to allow for transport from mother to young. Manipulation of these micelles has led to the incorporation of choice hydrophobic nutrients [22]. Vitamin D was dispensed to human volunteers within these casein nanoparticles, increasing vitamin D bioavailability in vivo, as casein particles undergo proteolytic cleavage in the stomach, releasing their encapsulated vitamins [21]. A similar strategy could be investigated to help producers assist neonates through weaning, a sensitive time for young as their digestive and immune systems are still maturing. In the absence of milk from the mother, weanlings must adapt to a complex carbohydrate diet and reduced immune support. This is an important stage not only for animal welfare reasons, but also from a production standpoint as weanlings that maintain their growth rates through the weaning process are healthier and heavier by slaughter [23]. Benefits from nutritional supplements may help weaned animals and poultry increase body size as well [24]. Nanoparticles engineered for nutrient delivery could facilitate this supplementation and bolster growth rates of livestock by increasing nutrient cargo bioavailability.

Orally administering additional nutrients to livestock via feed brings inherent challenges that must be considered when designing a carrier nanoparticle. Each compartment of the gastrointestinal tract has a unique environment that includes its own complement of enzymes and specific pH level. Nanoparticles must be able to overcome these obstacles to deliver their nutritional cargo at the appropriate location, i.e. the small intestine [25]. Akbari and Wu [26] investigated a nanoparticle made from the canola protein cruciferin, and demonstrated that these nanoparticles could encapsulate both hydrophobic and hydrophilic bioactive compounds, protect them from a simulated stomach environment, and release them in a simulated intestinal environment. Once nutrients are released from nanoparticles, they must reach the intestinal epithelia and carry out their function as if they were to have originated from the feed. The nanoparticle cannot remain in the intestine, as accumulation may prevent the absorption of other nutrients in the lumen. Cellular uptake, degradation, or removal from the body must occur. Depending on the type of nanoparticle, cellular uptake may not be preferred in the animal production industry where food safety must be considered. Cruciferin nanoparticles are digestible in the small intestine by the pancreatin enzyme, however, indigestible nanoparticle vehicles may be excreted naturally. Before entering the market, a thorough study of nanoparticle action must be done to test for potency and any unwanted biological consequences, like cytotoxicity.

While nutrient delivery at the nanoscale can occur naturally or synthetically, nanoparticles can stabilize bioactive compounds and assist in cellular uptake. To add a bioactive component directly to feed entails a risk of degradation and inaccessibility that can be countered through nanotechnological means. The small size of nanoparticles garners a heightened level of bioavailability compared to microparticles, particularly in the digestive tract, since nanoparticles can more readily pass through the intestinal mucosa. Huang et al. [27] used calcium carbonate and calcium citrate at the nano- and microscales to test bioavailability differences by measuring bone mineral densities of mice. Mice that were administered the calcium compounds at the nanoscale had denser bones compared to mice given micro-calcium and controls. It would be of interest to investigate whether delivering calcium in nanoform may help to strengthen bones of production animals such as turkeys, as body mass is heavily selected for over leg strength, making it difficult for turkeys to support themselves [28].

## Biocides

Nanoparticles may present a feasible alternative to antibiotics and may help bar pathogens from entering animal production sites. The unregulated use of antibiotics, a common practice in many countries, provides the impetus for bacteria to become drug-resistant. New legislation for the restriction of prophylactic antibiotic use in agriculture is gaining ground as a method to combat this growing problem [29]. Limiting antibiotic use necessitates the development of alternatives due to the high-density nature of modern animal production facilities which invites and expedites disease transmission [30]. Metal nanoparticles with net positive charges are drawn to negatively charged bacterial membranes, resulting in leakage and bacterial lysis [31]. Kim et al. [32] found that silver nanoparticles could inhibit the growth of hemorrhagic enteritis-inciting *E. coli* O157:H7 and yeast isolated from a case of bovine mastitis with an estimated MIC of 3.3-6.6 nmol/L and 6.6-13.2 nmol/L, respectively. At present, silver is used in medicine and dentistry to prevent wound infections and the formation of biofilms on catheters and dental appliances. Similarly, positively charged and/or quaternized polymeric nanoparticles can limit microbe growth [33]. Qi et al. [34] tested the antimicrobial activity of chitosan, a biopolymer with a positive surface charge (Zeta potential of approximately +51 mV), against a variety of bacterial pathogens and concluded that the MIC for all species tested was smaller than 0.25 μg/mL. Current biosecurity measures can impede pathogen entry but feed, water, air, and personnel may still serve as accession points [35]. The use of antimicrobial nanoparticles at these points may create additional hurdles for potential pathogens seeking entry.

Copper is regularly added to feeds for its ability to promote animal growth and performance in addition to its antimicrobial properties [36, 37]. As copper is delivered to animals orally in feed, the same challenges seen in nutrient delivery can be applied here. It has been reported that copper nanoparticles pass intestinal mucosa more easily than microforms, aiding in absorption. Gonzales-Eguia et al. [38] demonstrated that nanoform copper could better improve piglet energy and crude fat digestion through the augmentation of lipase and phospholipase A activity in the small intestine compared to a basal diet supplemented with CuSO_4_. These piglets experienced an increase in daily weight gain, metabolic rates, and immune capacity. Analysis of nanocopper influence on the immune system showed significantly greater total globulin and superoxide dismutase concentrations in blood serum, although hematology was unchanged among experimental groups. These results suggest that the nutrient value of feed can be enhanced through the addition of antimicrobial metal supplements in nanoform. However, further investigation is required before determining whether antibiotics in feed can be completely replaced by nano-antimicrobials.

Despite the expansion of antibiotic-resistance in bacteria, antibiotics have not yet been rendered totally ineffective against them. Their delivery and efficacy can be enhanced by nanoparticle carriers, potentially decreasing the dosage of antibiotics required for treatment. Susceptible bacteria lack at least one mechanism of resistance where resistant bacteria have, at minimum, one of the many known resistance mechanisms [39]. β-lactamases are resistance enzymes that cleave the β-lactam ring in β-lactam drugs such as penicillin and methicillin, destroying drug activity [40]. Loading penicillin onto polymeric polyacrylate nanoparticles has been identified as an effective method of making the β-lactam ring insusceptible to β-lactamase binding and cleavage, restoring penicillin function against methicillin-resistant *Staphylococcus aureus* (MRSA), a penicillin- and methicillin-resistant bacterial strain [41]. In a study by Turos et al., different penicillin-polyacrylate nanoparticle preparations were examined and it was found that two of them could inhibit MRSA just as well as the positive control, Penicillin G, and that one of the two had an MIC 8× lower than that of Penicillin G (2 μg/mL versus 16 μg/mL) [41]. No evidence of toxicity was seen during either the systemic or topical application of these nanoparticles in a murine model, and when applied topically to a dermal abrasion, the healing time was shortened by 3–5 d [42]. A similar study was performed using tetracycline-bound polymeric chitosan nanoparticles against a tetracycline-resistant *Escherichia coli* strain, requiring a minimum concentration of 700 μg/mL for inhibition of bacterial growth [43].

Nanoparticles made from natural materials that target Gram negative bacteria are most desired for feed, since bacterial pathogens are predominately Gram negative. This is due to the Gram-negative endotoxin, lipopolysaccharide (LPS), that is absent in Gram positive bacteria. It is important to monitor the materials provided to food animals as the bodily accumulation of toxins could pose a threat to consumers and potentially dampen public opinion towards the inclusion of nanotechnology in animal production, highlighting the importance of only selecting biocompatible nanoparticles for use in feed. However, the addition of nanoparticles in feed is not the only solution to limit livestock exposure to pathogens; the external application of hydrogels could assist in preventing pathogens from entering production facilities. Quaternization, which gives amine groups a permanent positive charge, of polymer nanoparticles such as poly(2-(dimethylamino)ethyl methacrylate) (qPDMAEMA) permits biocidal activity through electrostatic attraction between polymer and microbe, distorting the cell wall [33]. When qPDMAEMA is grafted onto agarose or cellulose nanocrystals, they can be made into hydrogels and used to cover surfaces to prevent bacterial adhesion, colonization, and biofilm formation. Their ability to bind viruses and virus-like particles has also been reported [44]. Moreau et al. [45] examined the biocompatibility of a few cationic polymers in vitro and in vivo and concluded that toxicity was dependent upon the molar mass of the polymer and the type of polycation. The unquaternized PDMAEMA (193.5 Da) was found to induce hemolysis and cause immediate mortality when administered intravenously in mice, indicating that its use as a biocide should be restricted to external purposes only, and should not be included in feed. Spreading biocidal hydrogels and nano-solutions across thresholds, pens, and feed containers presents yet another use that would benefit from further study [46].

## Meat and egg quality

The possibility of using nanoparticles to enhance meat and egg quality has also been investigated. For example, Wang and Xu [47] demonstrated that when finishing pigs destined for market were given chromium nanoparticles (200 μg/kg) in feed, they were 14.06% leaner at slaughter than control pigs fed a basic diet of corn-soybean meal. An increase in skeletal muscle mass and improved pork quality were achieved, with similar effects found when finishing pigs were fed chitosan nanoparticle supplements loaded with chromium [48, 49]. These chromium-loaded chitosan nanoparticles elevated the activity of hormone-sensitive lipase in adipose tissue while decreasing fatty acid synthase activity and boosting blood serum immune components [48, 49]. These data provide a compelling insight into the mechanism of action these nanoparticles have in pigs, and how they affect meat quality. Of further interest is the heightened chromium content in selected tissues, such as 184.11% in the longissimus muscle compared to the control, as some nanoparticles, i.e. Ag^+^ and Cu^2+^, have been found to cross the blood–brain barrier [47, 50].

The inclusion of nanomaterials in livestock feed or water can benefit the quality of product obtained, as well as the production cycle. Chromium nanoparticles added to poultry feed not only positively affected breast and thigh muscle protein content while simultaneously lowering cholesterol, but raised the average daily gain and feed efficiency of the broilers in the experimental group fed 500 μg/kg Cr^3+^ [51]. The implications of these results are shorter production cycles for better quality meat with less feed required to have broilers reach market weight. Conversely, when chromium nanoparticles were supplied to layers, there was no significant effect on body weight or egg production [52]. However, Sirirat et al. [52] did find that egg quality improved from higher chromium and calcium levels in the yolks and shell, respectively. Bioaccumulation of nanoparticles in the liver was noted for the experimental group, an observation shared with Chauke and Siebrits [53] in a study that replaced an antibiotic against coccidiosis with silver nanoparticles in water (0.083 mg/kg of silver compared to 0.001 mg/kg in the control). More information on the interplay between nanoparticle concentration and meat quality would be useful to ensure that quality is not sacrificed after long-term exposure [54]. The inclusion of nutrient supplements in livestock feed, regardless of particle size, will benefit the producer if there is still consumer demand for the final product. If meat and eggs obtained from an animal fed nanoparticle supplements are enhanced, or indiscernible from the original product, they are then likely to still be favourable to consumers. However, it is important to understand the role of the nanoparticle additive in a given biological system and by-products from that system to ensure it is safe for consumption before its application in animal production.

## Milk

Mastitis is an example of a common ailment among dairy cows with a variety of inciting factors, often bacterial, that can require the use of antibiotics to clear. Tilmicosin is an example of a drug used in mastitis cases that has negative side-effects if given at too high a concentration. In consideration of this, Han et al. [55] sought to control the release of tilmicosin by using hydrogenated castor oil-solid lipid nanoparticle carriers. Of concern was what the extended half-life of the therapeutic would mean for milk discard times, as tilmicosin was present in mouse blood serum for 5 h without nanocarrier delivery, and 8 d with nanocarrier delivery [55]. However, a lower dosage was required for resolution in a *S. aureus*-induced murine mastitis model (10 mg/kg versus 20 mg/kg) [56]. Careful manipulation of therapeutic nanocarriers to find a balance between dosage and half-life could serve to benefit producers by minimizing milk discard times and the amount of milk wasted.

Nanotechnology can also help to ensure that the quality of milk is safe for human consumption through novel foodborne pathogen detection techniques. Sung et al. [57] developed nanocomposites containing anti-*S. aureus* antibodies, gold nanoparticles, and magnetic nanoparticles to provide a 40 min colorimetric test for the presence of *S. aureus* in milk. An interesting feature of these nanocomposites is the antibody, whose specificity and selectivity could be modified to capture a variety of pathogens [57]. Wang et al. [58] demonstrated a similar technique, employing polyclonal antibodies and gold nanoparticle immunochromatographic strips to detect toxins present in milk within 10 min, using the carcinogenic aflatoxin M1 as an example. While a large focus has been to remove potentially harmful contaminants from milk, there has also been some interest in mixing nanoparticle supplements directly into cow’s milk for human consumption. Lee et al. [59] combined nanopowdered oyster shell into milk with the intention of increasing the calcium content from 100 to 120 mg/mL to a level more suitable for growing children and post-menopausal women. Supplementing milk with calcium from nanopowdered oyster shell did not negatively alter its sensory or physicochemical qualities after 16 d of storage at 4 °C [59].

## Veterinary medicine

Nanomedicine is an intriguing discipline in nanotechnology that is showing progress in both diagnostics and therapeutics. Metallic and nanostructured particles are useful diagnostic tools in biomedical research that can be used to visualize the status of a cell or drug distribution in the body. Magnetic nanoform metals, i.e. iron oxide, can be taken up by cells and imaged in vivo at high concentrations using magnetic resonance imaging (MRI) [60]. Nanostructured particles can be made to fluoresce through light activation or two-photon excitation [61, 62]. Further to these diagnostic nanoparticles, exciting developments have been made in molecular-based lab-on-a-chip technologies for qualitative and quantitative biological analyses. Requiring small volumes of analyte and reagents, producing little waste, and shortening wait times make these lab-on-a-chip technologies an attractive option [63]. There are currently a number of products functioning at the microscale available on the market with nanoscale products just emerging; see Tian et al. [64] for more information.

Drug delivery can be monitored through fluorescent nano-carriers. For example, light activated, fluorescent nanostructured glucose- and sucrose-derived nanoparticles can be used to monitor the localization of bound chemotherapies [61]. The biocompatibility of carbohydrate-derived nanoparticles has been demonstrated in a human lung carcinoma cell line by Ajmal et al. [61]. Their findings showed that upon binding methotrexate, a chemotherapeutic drug, the conjugate nanoparticles were reported to have a cytotoxicity close to that of cells treated only with methotrexate. However, the advantage of delivering this drug with a nanoparticle that fluoresces after light activation is the ability to trace the drug. For even better tracking, using a carrier nanoparticle capable of being activated via two-photon excitation can provide a 3D spatial image over a greater tissue depth than a particle activated with visible UV light [62, 65]. As chemotherapies are typically delivered in a high dose regimen, the ability to observe their distribution in the body through fluorescence could help to reduce off-target side effects by better targeting them to desired areas.

Fluorescence is not a shared trait among all nanoparticle drug carriers, and their mechanisms of drug binding and release can be quite varied, especially amongst nanostructured particles. Cylindrical nanotubes can trap pharmaceutical agents within an internal matrix surrounded by an outer layer of poly (L-lactide) or poly (D-lactide) [66]. When these enantiomers come together in solution, they interact with each other to reconfigure their structures from cylinders to spheres, releasing trapped materials in the process. There is no requirement for external stimuli for drug release as there is in some light activated nanoparticles, only that the two nanotubes interact. Other nanostructured particles can be self-loading like albumin-dextran nanoparticles with hydrophobic drugs [67]. Albumin from bovine serum can be stabilized with dextran in aqueous solutions and can bind medicine through hydrophobic and electrostatic interactions. When tested with ibuprofen, albumin-dextran nanoparticles could take up 0.7-unit weight of ibuprofen per 1-unit weight of the conjugated particle [67]. These nanostructured materials present new mechanisms for pharmaceutical uptake and release in nanomedicine, potentially serving as methods to increase release specificity and reduce lag times between drug delivery and effect in the future.

## Reproduction

Animal production revolves around animals meant for slaughter. Finishing livestock are the offspring of individuals intended for breeding who have highly ranked genotypes and phenotypes. The traits and reproductive abilities of breeders gives them high value. Some nanoparticles have been demonstrated to enhance fertility and protect spermatozoa through the functional groups they carry. Artificial insemination is widely preferred in animal production as an alternative to live cover strategies due to the lower risk for animals and producers. Commonly done to diversify genetic backgrounds and boost selection of livestock traits, artificial insemination has the potential to be enhanced through the integration of nano-techniques such as the non-invasive bioimaging of gametes, nanopurification, and protectants in cryopreservation.

In order to optimize the efficiency of artificial insemination, livestock gamete biology and reproductive obstacles to fertilization must first be elucidated. Recently, quantum dots have been explored as a research method to improve understanding of mammalian spermatozoon and oocyte movement and their interactions in a physiological setting. These self-illuminating, inorganic nanoparticles are of interest to the field of theriogenology as they are biocompatible, photo-stable, and have a greater signal intensity than organic fluorescent molecules previously used to image gametes and other cell types in vivo [68–70]. Feugang et al. [68, 71] have demonstrated the real-time tracking ability of bioluminescent resonance energy transfer-conjugated quantum dot (BRET-QD) nanoparticles in vitro, in situ, and ex vivo using pig male gametes (*Sus scrofa domesticus*). Quantum dots can provide targeted or non-targeted imaging as a function of their size, emitted wavelengths, and conjugation possibilities [68, 71]. This engineered nano particle provides a new mean to visualize the molecular and cellular events during fertilization, in a similar way to fluorescent proteins, but at greater tissue depths [71, 72]. Signal strength of quantum dots are dose-dependent and a higher concentration may be required for in vivo imaging on larger animals. Thus, the composition of quantum dots should be further optimized for biocompatibility as many of the current ones include heavy metals, such as cadmium and lead, which may be cytotoxic at high levels [73]. However, if quantum dot concentrations and surface chemistries are carefully selected, cytotoxicity may be decreased or eliminated [74].

Nanopurification of semen can be used to separate damaged sperm from undamaged, healthy sperm. One method is to coat magnetic nanoparticles with antibodies against ubiquitin, a surface marker of defective sperm, for a protein-based removal strategy [75]. A lectin-based strategy features magnetic nanoparticles coated with lectins that bind glycan exposed at the surface of the sperm through acrosomal damage [76]. Nanopurified bull spermatozoa (*Bos taurus*) achieved conception rates equal to those of unpurified semen at half the concentration with no negative impacts reported for inseminated cows or calves [75]. Thus, more females can be inseminated from one sample of nanopurified, diluted ejaculate. Further identification of spermatozoa biomarkers will allow for increased selection ability and fertility improvement, as targeted through the antibody or lectin strategies.

Cryopreservation of sperm can be enhanced by turning to nano-protectant additives in extenders. Used to dilute sperm, extenders are buffering agents and provide sperm with nutrients required for prolonged storage. They serve to protect and contain antibiotics, to prevent bacterial growth from affecting sperm quality and infecting inseminated females [77]. Antimicrobial nanoparticles may serve to replace extender antibiotics in the future as some antibiotics have been shown to inhibit sperm motility and viability in a dose-dependent manner [78]. Nanoparticles may also facilitate the addition of natural products in extenders to increase sperm motility. Research groups have reported that the addition of honey, sugarcane juice, tomato juice, and pineapple juice can increase the survivability of sperm stored at room temperature [76, 79]. While nanoparticles were not involved in those studies, it would be interesting to know how sperm quality would be effected if the functional groups of each product were to be delivered via nanoparticle. As sperm can be shipped internationally over multiple days, extenders with a higher capacity for preserving samples undergoing freeze-thaw cycles would be beneficial [80].

Further advancements in reproductive biotechnology may be possible with the greater inclusion of nanoparticles in molecular biology techniques. Sperm-mediated gene transfer is onesuch approach where mesoporous silica nanoparticles can be loaded with nucleic acids and proteins [81]. These nanoparticles can form strong associations with spermatozoa in vitro, and do not have any diminutive effects on sperm function or quality. Transfections with polymeric nanoparticles, such as PDMAEMA, chitosan, and polyethylenimine, have been reported to be advantageous over traditional viral approaches provided low concentrations of polymers are used [82]. The molecular weight of the nanopolymer has great influence over transfection efficacy and toxicity, i.e. the optimal molecular weight for transfection with PDMAEMA has been determined to be 60 kDa [82].

With continued exploration and refinement, nanoparticles could play a significant role in animal reproduction. However, it should be noted that some nanoparticles are spermatotoxic which may have serious consequences if breeder reproduction is affected. Zinc oxide and titanium oxide nanoparticles are two examples that reduce in vitro sperm viability in a dose- and time-dependent manner by membrane weakening and DNA fragmentation [83, 84]. Barkhordari et al. [83] incubated human sperm with zinc oxide nanoparticles and found that a concentration of 500 μg/mL would significantly increase cell death after 45 min, while a concentration of 100 μg/mL would significantly increase cell death after 180 min. Pawar and Kaul [84] found that buffalo sperm (*Bubalus bubalis*) incubated with 100 μg/mL of titanium oxide nanoparticles would have reduced viability. At 10 μg/mL titanium oxide was found to prematurely increase sperm capacitation, which is the final required step in sperm maturation for oocyte penetration and fertilization. While nanoparticles may be points of advancement for the animal production industry, precautions should also be taken when considering the employment of nanoparticles for assisting reproduction.

## Future prospects

As nanotechnology continues to develop and garner more attention, its applications in the animal production industry will become more expansive. The regular inclusion of nano-supplements to fortify livestock feed is likely possible in the near future; however, it will take longer for nanoparticles to fully replace antibiotics in feed as many biocidal candidates must still be tested in vivo before undergoing clinical trials and food safety tests in accordance with government regulations. External uses for nanoparticles have already been integrated into some aspects of animal production, i.e. antiseptic wound dressings, and more are to follow [85, 86]. For studies interested in nanoparticles with anti-cancer properties, it is important to investigate nanoparticle cytotoxicity in both cancer cell lines and normal, healthy cell lines. Only using cancer cells and claiming the nanoparticle under investigation has anti-cancer properties may be misleading, as the nanoparticle may be cytotoxic to all cell types. In vivo studies are needed for verification of nanoparticle functions seen in in vitro research. Table 3 outlines nanoparticle experiments relevant to the animal production industry and identifies gaps in knowledge where future research will be required.

**Table 3:** Summary of nanoparticle studies relevant to animal production

Nanoparticle	Type	Experiment	In vitro/ In vivo	Cell line	Animal production application	Reference
Gold and Copper	Metal	Biocides for water treatment	In vitro	N/A	Biocide	[5]
Casein micelles	Natural	Determining storage capacity and stabilization of encapsulated bioactive compounds and their bioavailability	In vivo Humans	N/A	Nutrient delivery	[21]
Lipid nanoparticles	Nanostructured	Simulated digestion assay to test bioavailability of loaded compounds	In vitro	N/A	Nutrient delivery	[25]
Cruciferin	Nanostructured	Encapsulation abilities and nutrient release studies	In vitro	Caco-2 (Human cancer cells)	Nutrient delivery	[26]
Calcium carbonate and calcium citrate	Metal	Bioavailability differences between microparticles and nanoparticles	In vivo Ovariectomized mice	N/A	Nutrient delivery	[27]
Silver	Metal	Eco-friendly biocide synthesis	In vitro *Vibrio cholerae*	N/A	Biocide	[31]
qPDMAEMA-agarose	Polymer	Microbial growth inhibitory properties of qPDMAEMA in solution and hydrogels	In vitro *E. coli* *S. aureus* Biofilms	N/A	Biocide	[33]
Copper	Metal	Enhancing growth promoting effects of copper by nanoscaling	In vivo Piglets	N/A	Nutrient delivery and Biocide	[38]
Gold	Metal	Functionalize with amoxicillin to overcome bacterial resistance.	In vitro Gram + Gram – In vivo Mice	L929 (Mouse fibroblasts)	Biocide	[40]
Polyacrylate	Polymer	Testing protective abilities towards loaded penicillin and aiding its antibacterial activity	In vitro *S. aureus* (methicillin-susceptible and -resistant strains)	N/A	Biocide	[41]
Chitosan	Polymer	Evaluating efficiency of drug loading and release	In vitro *E. coli* (tetracycline-resistant strain)	N/A	Biocide	[43]
qPDMAEMA-CNC	Polymer	Analyzing viral-binding ability for the concentration and extraction of viruses and virus-like particles	In vitro Cowpea chlorotic mottle virus Norovirus-like particles	*Spodoptera frugiperda* Sf9 (Insect cells)	Biocide	[44]
Triclosan	Polymer	Increasing antimicrobial activity of organic agents through aqueous nanodisperive techniques	In vitro *Corynebacterium*	N/A	Biocide	[46]
Iron oxide	Metal	Imaging applications in functional studies in vivo	In vitro	Neural progenitor cells Pheochromocytoma cells (Rat lineage)	Veterinary Medicine	[60]
Carbon (glucose- and sucrose-derived)	Nanostructured	Demonstrating anticancer bioactivity of loaded drugs	In vitro	H157 (Human cancer cells)	Veterinary Medicine	[61]
Mesoporous silica	Nanostructured	Spatial imaging of drug release in the body	In vitro	MCF-7 (Human cancer cells)	Veterinary Medicine	[62]
Poly(L-lactide)- and Poly(D-lactide)-b-poly(acrylic acid)	Nanostructured	Investigation into new controlled delivery of therapeutics	In vitro	N/A	Veterinary Medicine	[66]
Albumin-dextran	Nanostructured	Bind hydrophobic drugs to create aqueous solutions	In vitro	N/A	Veterinary Medicine	[67]
Zinc oxide	Metal	Toxic effects on livestock sperm	In vitro	Sperm (Human)	Reproduction	[83]
Titanium oxide	Metal	Toxic effects on livestock sperm	In vitro	Sperm (*Bubalus bubalis*)	Reproduction	[84]
Antibody-coated or lectin-coated F_2_O_3_	Metal	Nanopurification of semen	In vitro	Sperm (*Bos Taurus*)	Reproduction	[75]
Mesoporous silica	Nanostructured	Transfer mediator for nucleic acid/protein cargo to sperm	In vitro	Sperm (Boar)	Reproduction	[81]
Silver	Metal	Antimicrobial testing of silver nanoparticles bound to cellulose fibers with alkali lignin	In vitro *E. coli*	N/A	Biocide and Veterinary Medicine	[85]
Biocellulose	Natural	Designing an antiseptic, collagen-stimulating wound dressing	In vitro Infectious bacteria	N/A	Biocide and Veterinary Medicine	[86]

## Conclusions

There are many applications for nanoparticles in animal production and this review serves to highlight these uses and to identify potential opportunities for future applications. Nanoparticles are already available on the market and, with continued development, their properties will be more finely optimized for a wider selection of applications. The use of nanotechnology in animal production is still in its infancy but encouraging results from nutrition, biocidal, remedial, and reproductive studies are driving further investigation.

## Abbreviations

BRET-QD: Bioluminescent resonance energy transfer-conjugated quantum dot
LPS: Lipopolysaccharide
MIC: Minimum inhibitory concentration
MRI: Magnetic resonance imaging
MRSA: Methicillin-resistant *Staphylococcus aureus*
PDMAEMA: Poly(2-(dimethylamino)ethyl methacrylate)
qPDMAEMA: Quaternized poly(2-(dimethylamino)ethyl methacrylate)
